# Health Tract, No. 151

**Published:** 1863-07

**Authors:** 


### HEALTH TRACT, No. 151.

### CHOLERA,

Or "Asiatic Cholera," as first known in this country in 1832 and '33, is chiefly a disease prevailing in warm weather, or rather in a warm atmosphere, for it can be created at any season, and in the coldest latitudes, by combining the proper degrees of the three essential requisites, to wit, moisture, vegetable decay, and a regular heat, exceeding eighty degrees. The great and distinguishing feature of cholera is a copious, frequent, and painless discharge from the bowels of a substance almost as thin as water, with a whitish tinge, as if rice had been washed in it, or as if a little milk had been dropped in it. When this occurs the patient soon begins to perspire profusely, the skin assumes a leaden hue and shrivels up, the nails become blue, insufferable cramps come on, and the victim's death occurs in a few hours with the most perfect calmness, in the fullest possession of all the faculties, and absolute freedom from every pain.

Three things ought to be known, in reference to cholera, by every human being:

First: The writer has never known a case in which it was not preceded, for one, two, or more days, by the bowels acting twice, or oftener, in every twenty-four hours; universally styled "the premonitory symptoms."

Second: A cure is impossible under any conceivable circumstances, without absolute quietude of body, on a bed, for days together; the time of confinement being shortened, in proportion to the promptitude with which the quietude is secured, after the first action of the bowels has taken place, which gives a feeling of tiredness, and, on sitting down, a sensation of rest and satisfaction.

Third: When the patient ceases to urinate he begins to die, and its resumption is a certain index of recovering health, always and infallibly.

One of the usual attendants of an attack of cholera is an unconquerable tendency to vomit. The very instant any thing reaches the stomach, even if it is but cold water, it is ejected; the mildest food meets the same fate in such cases, much less will medicine find a lodgment, except one, and that it is impossible to vomit up if it once reaches its destination; that medicine has no taste, it is small in bulk, will retain its virtues for a quarter of a century, as the writer knows by personal experience and repeated observation. Unless it is in the very last stages, it is believed capable of arresting the disease in nine cases out of ten—a pill made up of ten grains of calomel with a little gum-water; if the symptoms do not abate in two hours, double the dose, and let it work itself off; do nothing else, but let the patient be quiet and eat all the ice he can possibly want.

